# A Reply to Dr. J. M. Thomas

**Published:** 1893-11

**Authors:** C. Evans Johnson

**Affiliations:** Atlanta, Ga.


### A REPLY TO DR. J. M. THOMAS.

EDITOR SOUTHERN MEDICAL RECORD:

My attention has been called to an article contained in your October issue entitled, "A Plagiarism Exposed" from the pen of Dr. J. M. Thomas, of Atlanta, who made his entrance into the profession only a few months ago.

It was my original intention to give no notice to said article, but being urged to do so by numerous friends, have decided to call attention to a few points regarding it. First, being requested in person by the Secretary of the Georgia Medical Association to prepare a paper for the Americus meeting and having a limited amount of time to do so, owing to the close proximity of the date of assembling of such Association, decided to give simply a *resume* of the work and investigation in the line of "Sterility in the Male," a subject in which I have long been interested. Seeing later that it would be impossible for me to attend the meeting, I cast aside the notes made and gave the matter no further thought. Some thirty days after meeting of the Association, I received a postal card from the Secretary requesting that paper be sent him for insertion in Transactions. I hastily prepared same and sent to him, a casual examination of which will disclose the fact that no claim is made to any original research in this line by myself, but upon its face shows itself to be simply a review of the investigation by others, principally Gross and Ultzmann, proper reference being made to them in the outset, a fact which the critic ignores, and states that he was greatly puzzled as to "the source of the mysterious paragraphs."

It is wholly unnecessary for me to say more, but I desire to express the hope that every one who may be interested in the matter will give my articie a close examination, it purporting to be nothing more than the aggregation of views of others upon the subject, with comments by myself.

C. EVANS JOHNSON, M. D., Atlanta, Ga.

